# Identification and Use of Assessment Tools in Spanish Occupational Therapists: An Exploratory Study

**DOI:** 10.3390/healthcare10101902

**Published:** 2022-09-28

**Authors:** Daniel Prieto-Botella, Alicia Sánchez-Pérez, Sergio Sánchez-Moreno, Miriam Hurtado-Pomares, Paula Peral-Gómez, Cristina Espinosa-Sempere, Iris Juárez-Leal, Paula Fernández-Pires, Desirée Valera-Gran, Eva-María Navarrete-Muñoz

**Affiliations:** 1 Department of Surgery and Pathology, Miguel Hernández University, 03550 Alicante, Spain; 2 Grupo de Investigación en Terapia Ocupacional (InTeO), Miguel Hernández University, 03550 Alicante, Spain; 3 Alicante Institute for Health and Biomedical Research (ISABIAL-FISABIO Foundation), 03010 Alicante, Spain

**Keywords:** assessment tools, occupational therapy, evidence-based practice

## Abstract

The use of standardized assessment tools is essential for the occupational therapy intervention process to ensure clinical practice is appropriate and of high quality. This study aimed to explore which assessment tools Spanish occupational therapists use in their clinical practice. An ad hoc online questionnaire of 19 open and closed questions was used to collect information on assessment tools, socio-demographics, and academic background. The assessment tools were classified according to the main domains of occupational therapy using the last edition of the American Occupational Therapy Association framework. The survey was completed by 73 Spanish occupational therapists, of whom 86.3% were women; the median age was 31 years, and more than 60% treated people suffering from neurological and neurodegenerative disorders or worked in geriatric medicine. Of 147 assessment tools identified, those designed to assess activities of daily living, body functions, process skills, and motor skills were the instruments most frequently used. Specifically, the Barthel Index, Lawton Instrumental ADL Scale, Functional Independence Measure, Sensory Profile 2, and Mini-Examen Cognoscitivo were the tools most employed by Spanish occupational therapists. However, more than one third of the participants used assessment tools without knowing whether they were validated in the Spanish context and/or a specific target population. To our knowledge, this is the first time a study has examined the use and type of assessment tools in Spanish occupational therapists. Our results may constitute a positive step forward for developing strategies for enhancing evidence-based assessment in occupational therapy practice.

## 1. Introduction

Assessment is a key element in the occupational therapy intervention process. Conducted at the beginning to design an intervention, during the process, to tailor the intervention procedures, and at the end of the process, to evaluate the results obtained from the intervention, it provides the necessary information to ensure adequate clinical practice [1,2]. An appropriate assessment requires standardised tools to provide accurate, objective and reproducible data. In scientific terms, this also allows health professionals to quantify progress and ensure clear and precise reporting of clinical results [3]. Moreover, in terms of healthcare delivery, using standardised assessment tools contributes to evidence-based practice [4,5], which supports healthcare practitioners in making clinical decisions for optimal patient care and safety [6,7].

The development, cross-cultural adaptation, and psychometric evaluation of standardised tools is a costly and time-consuming process that requires the collaboration of staff with expertise in translation and statistical analysis [8]. The need for specific prior training in the design, adaptation and/or validation of clinical assessment instruments is undoubtedly a challenge that limits potential collaboration of healthcare personnel, such as occupational therapists, in this process. A review of standardised instruments in occupational therapy practice recently published by Romli and colleagues [9] indicated that, of the 641 occupational-based instruments found, only 7% of the tools were developed with the help of an occupational therapist. Despite this, assessment tools in clinical practice are an area of growing interest among occupational therapy professionals, especially in Spain. In the last few years, a great deal of research has focused on the development and evaluation of the psychometric properties of various tools commonly used in the occupational therapy field, most of which are the result of work carried out in doctoral theses [10,11,12,13,14,15].

To date, several studies have been conducted to identify standardised assessment tools used by occupational therapists in different clinical areas, such as cognitive disability [16,17,18], mental health [19], geriatrics [20] and acute physical injury [21]. In addition, the use of assessment tools in occupational therapy practice has been explored in Anglo-Saxon countries, such as the United States, Australia, Canada, and New Zealand [16,21,22,23,24,25]. However, these studies have shown that around 10–30% of occupational therapists surveyed did not use standardised assessment tools in their daily clinical practice [16,17,18,19,20,21,22,23]. Moreover, some studies have raised a particular concern that the most widely used assessment tools in occupational therapy often have not undergone a formal process of validation in the context and/or population in which they are being used [17,21]. Since assessment plays a crucial role in the clinical decision-making process and in healthcare intervention design, monitoring the use of validated assessment tools is essential to ensure high-quality healthcare delivery and patient safety. However, to our knowledge, no study has examined which assessment tools occupational therapists use in Spain. Therefore, this study aimed to identify assessment tools used by Spanish occupational therapists in their clinical practice. Secondarily, the occupational therapists were asked if they knew whether the assessment tools they used were validated in the Spanish population. Finally, the identified assessment tools were classified according to the main domains of occupational therapy assessed using the fourth edition of the Occupational Therapy Practice Framework (OTPF-4) [26] of the American Occupational Therapy Association (AOTA).

## 2. Materials and Methods

### 2.1. Study Design

An exploratory study was conducted through an online survey developed on the Google Forms platform according to the criteria proposed by the Checklist for Reporting Results of Internet E-Surveys (CHERRIES) [27]. A convenience sampling was used to recruit potential participants through a dissemination campaign via social networks, such as Twitter, LinkedIn, Facebook, Instagram, Telegram and WhatsApp. Additionally, collaboration was requested via e-mail from occupational therapy professional associations, universities offering occupational therapy degrees, and occupational therapists. The data collection was performed from February to July 2019. The option in Google Forms, “limit to one response”, was used to avoid duplicate responses. Moreover, during the recruitment campaigns, it was clearly explained that participants had to be occupational therapy graduates or diploma holders, thus excluding any survey that occupational therapy students answered. A total sample of 73 occupational therapists completed the survey.

### 2.2. Study Variables

Study information was collected using an ad hoc online questionnaire containing 16 open and closed questions divided into three sections: assessment tools, socio-demographic data, and academic background. An expert panel comprised of five academic researchers (D.P.-B, M.H.-P., I.J.-L., D.V.-G, E.-M.N.-M.), three of whom were occupational therapists with extensive clinical experience, developed the questionnaire. Since our main interest was to collect as much information as possible about the assessment tools Spanish occupational therapists used in their clinical practice, we used open questions for this part of the questionnaire. As a proxy for assessing how occupational therapists implement evidence-based practice, we also included some questions about their knowledge of adapted and validated tools for the Spanish population. Information about socio-demographic data and academic background was obtained through closed-ended questions to facilitate analysis of the results. Before starting the study, the questionnaire was pilot-tested with an occupational therapist who worked as a clinician (S.S.-M.). All answers were reviewed and discussed by the expert panel to determine the definitive version of the questionnaire (available in the Supplementary Materials S1).

#### 2.2.1. Assessment Tools

Participants were requested to indicate the assessment tools they used in their routine practice setting and other assessment instruments they knew. In this study, we only analysed the information regarding the measures participants reported using in their daily clinical practice. Moreover, participants were asked to indicate whether the reported tools were validated in the Spanish population. Both types of questions were presented in an open-ended format. After collecting all the information, the assessment tools were classified according to the main domains of occupational therapy (e.g., Occupations: activities of daily living, instrumental activities of daily living, health management, rest and sleep, education, work, play, leisure, social participation; Contexts: environmental factors, personal factors; Performance patterns: habits, routines, rituals, roles; Performance skills: motor skills, process skills, social interaction skills; Client factors: values, beliefs, and spirituality, body functions, body structures) based on the OTPF-4 [26]. These domains were not exclusive, and the same assessment tools could be classified into different domains if necessary. All the assessment tools were randomly distributed between D.P.-B., A.S.-P., S.S.-M., H.H.-P., P.P.-G., C.E.-S., I.J.-L., and P.F.-P. for peer-review screening. Several measures did not fit into any domain because these tools measured the potential outcomes of occupational therapy intervention. Following the elements of the occupational therapy process included in the OTPF-4 [26], we created an additional category (Outcomes: quality of life, health and wellness, wellbeing) to classify the remaining assessment tools. Discrepancies between the authors were resolved through consultation with D.V.-G. and E.-M.N.-M. Responses regarding the validity of the assessment tools were coded manually by D.P.-B. and E.-M.N.-M. and categorised as “validated”, “not sure”, or “do not know”.

#### 2.2.2. Socio-Demographic Information

The following socio-demographic features of participants in the study were also collected: age, sex (female, male), occupational therapy degree (3-year bachelor’s degree, 4-year bachelor’s degree), master’s degree (yes, no), employment status (self-employed, employed, unemployed, other), weekly working hours, workplace (private clinic, public health centre, foundation/association, client’s home, university, others), and practice setting (including neurology, dementia, geriatrics, early childhood intervention, mental health, community care, intellectual disability, school, academic teaching/research, traumatology/rheumatology, others).

#### 2.2.3. Academic Background

The questionnaire also gathered information about the academic background of participants. For example, they were asked to indicate the university where they were trained as occupational therapists, the type of qualification (degree, diploma), the year of completion of the degree/diploma, and whether they had completed a master’s degree (yes, no).

### 2.3. Statistical Analysis

The results from the survey were transcribed into an Excel database and analysed using the statistical software R version 4.0.0 (R foundation for statistical computing, Vienna, Austria, https://www.r-project.org/). The normality of quantitative variables was tested using the Kolmogorov–Smirnov test. For descriptive analysis, we described qualitative variables by frequency and percentage (%). Depending on whether the data followed a normal or non-normal distribution, quantitative variables were described using the mean and standard deviation (SD) or the median and interquartile range (IQR), respectively.

### 2.4. Ethical Considerations

This study followed a non-experimental design in which information was collected anonymously, so ethics committee approval was not required. This research complied with the requirements of the International Ethical Guidelines for Health-related Research Involving Humans (https://cioms.ch/wp-content/uploads/2017/01/WEB-CIOMS-EthicalGuidelines.pdf, accessed on 28 January 2019), prepared by the Council for International Organizations of Medical Sciences in collaboration with the World Health Organization (WHO), which indicate that some studies may be exempt from approval by an ethics committee when the data for the study are generated by observation, and when data that could identify individual persons or groups are anonymised or coded (Guideline 23. Requirements for establishing research ethics committees and for their review of protocols, available online at: https://cioms.ch/wp-content/uploads/2017/01/WEB-CIOMS-EthicalGuidelines.pdf, p. 90, accessed on 28 January 2019). In addition, the present research followed the ethical principles formulated by the Declaration of Helsinki. The confidentiality and anonymity of the participants were preserved, as stipulated in Organic Law 3/2018, of 5 December, on the Protection of Personal Data and the guarantee of digital rights.

## 3. Results

### 3.1. General Characteristics of the Study Participants

Table 1 shows the general socio-demographic features of participants in this study. The survey was completed by 73 Spanish occupational therapists, of whom 86.3% were women; the median age was 31 (IQR: 26–37) years. More than half of the participants (52.2%) obtained qualifications in occupational therapy at diploma level (i.e., 3-year bachelor’s degree), a third of whom had completed their studies before 2009. In addition, more than two-thirds of the participants (69.1%) had completed a master’s degree. Regarding work-related features, for the most part, participants reported working as occupational therapists (93.2%) at the time of answering the survey, 15.1% of whom were self-employed. Overall, they worked a median of 34 (IQR: 20–40) working hours per week, mostly in private clinics (38.4%) and foundations/associations (24.7%). The areas of neurology (38.4%), early intervention (12.3%), dementias (12.3%) and geriatrics (11.1%) were the practice settings in which participants worked. The total number of assessment tools for occupational therapy identified in the study are available in the Supplementary Materials, S1.

### 3.2. Assessment Tools for Occupational Therapy

In this study, 147 assessment tools were identified, of which the participants recognised 76 (51.7%) as validated for use. Table 2 displays the percentage use of the assessment tools reported by the participants according to the main domains of occupational therapy. Overall, the assessment tools specifically designed to measure occupations were used by almost all the participants, of which 76.7% habitually used instruments for activities of daily living (ADL) and 61.6% for instrumental activities of daily living (IADL). The instruments for measuring performance skills were also routinely applied by a considerable proportion of the participants. Tools for the assessment of process skills were used by 78.1% of the occupational therapists. Around two-thirds frequently used measures of motor skills (64.4%), and more than half used instruments for social interaction skills (56.2%). Moreover, most occupational therapists reported applying measures designed to assess body functions (84.9%). The assessment tools less frequently used by the participants (11.0%) were those aimed at assessing performance patterns, such as rituals and roles, and contexts, including both environmental and personal factors. Beyond the domains of occupational therapy, there was a low percentage of instruments used for evaluating outcomes, including quality of life, health and wellness, and well-being measures.

According to each participant’s response, 68.5% of occupational therapists perceived that the assessment tools they used in their clinical practice were validated, while around a third (31.5%) reported having doubts (12.4%) or did not know (15.1%). Table 3 shows the list of the ten assessment tools most used by the participants in this study. The Barthel Index (50.7%) was the most frequently used assessment instrument, followed by the Lawton Instrumental ADL Scale (39.7%), Functional Independence Measure (FIM, 21.9%), Sensory Profile 2 (20.5%) and the Mini-Examen Cognoscitivo (MEC, 15.1%), i.e., the Spanish version of the Mini-Mental State Examination. Among these instruments, 72.6% were used for assessing ADL, 60.2% for cognitive skills, 57.6% for motor and praxis skills, 39.7% for IADL, 34.2% for sensory-perceptual skills, and 20.5% for emotional regulation skills. Regarding the validity of these tools, the Barthel Index (81.8%), FIM (81.2%), Mini-Examen Cognoscitivo (81.8%), and Nine-Hole Peg Test (87.5%) were the tools most often recognised as validated by those that reported using them in their clinical practice. Conversely, among the ten assessment tools most frequently used, the Sensory Profile 2, Sensory Integration and Praxis Test, and Tinetti Test were the tools least often recognised as validated.

## 4. Discussion

This study identified 147 assessment tools that Spanish occupational therapists use in their clinical practice. In classifying the tools according to the domains of occupational therapy, we observed that, for the most part, Spanish occupational therapists used these instruments to assess occupations, including ADL and IADL, body functions, and process skills. However, the use of measures of motor skills was also considerable. More specifically, the results from the survey showed that the Barthel Index, Lawton Instrumental ADL Scale, FIM, Sensory Profile 2 and MEC were the most frequently employed tools in occupational therapy interventions. In the context of clinical decision-making, using reliable and valid assessment instruments is essential to ensure the quality of clinical assessment by adding a measure of objectivity to clinical reasoning, thereby improving the quality of decision-making. However, in this study, it was observed that almost one-third of the participants used assessment tools without knowing whether they were validated in the Spanish context and/or with a specific target population. Although a wide range of tools for clinical assessment in occupational therapy was identified, it was also observed that only a small number of the measures were habitually used in practice. The results of this study showed that instruments for measuring ADL, IADL, body functions, process skills, and motor skills were among the most used assessment tools in current occupational therapy practice in Spain. Although the assessment tools classified into the body functions and process skills occupational therapy practice domains include a broad spectrum of instruments, there were many specific instruments for assessing mental functions and cognitive skills (Supplementary Materials S1).

The results of this study partly coincide with those of previous studies conducted in Canada and Australia, where occupational therapists used, in large part, tools to assess cognitive skills and ADL. Moreover, the present results align with a survey conducted in 2012 with 794 occupational therapy practitioners from the United States [22]. According to this study, the assessment measures used most frequently were those evaluating motor functions. However, our study showed instruments for assessing cognitive functions, such as the Mini-Mental State Examen, or sensory-perceptual skills, such as the Sensory Profile, were also reported [22]. In this latter study, the Peabody Developmental Motor Scale and the Beery–Buktencia Test of Visual Motor Integration, generally used in children, were the motor measures most frequently used. In contrast, our study found that the motor measures most widely used by the occupational therapists surveyed were the Nine-Hole Peg Test and the Tinetti Test, which are assessment tools aimed at older people or adults with neurological diseases. This difference, may be explained by the fact that more than 50% of the occupational therapists participating in the study by Piernik-Yoder and Beck (2012) worked with paediatric populations, especially in school settings. In contrast, more than 60% of the participants in our study treated people suffering from neurological and neurodegenerative disorders or worked in geriatrics assisting elderly people at risk of chronic disease or disability. In this respect, it is recognised that the field of neurology is an area where cognitive, physical, and functional aspects are assessed to a greater extent [28,29]. This may explain why functional assessments and instruments for measuring cognitive or motor impairment were reported in higher proportions by the occupational therapists who participated in our study. On the other hand, regarding the lower use of instruments for measuring contexts or performance patterns, such as roles or rituals, our results reflect the lack of evidence on using these assessment tools as a basis for clinical decision-making. Since these measures require eliciting a large amount of subjective content, it is probable that occupational therapists do not assess them using formal procedures, but, instead, by using non-standardised observations [9].

The Barthel Index, Lawton Instrumental ADL Scale, FIM, Sensory Profile 2 and MEC were the assessment tools most frequently used by the Spanish occupational therapists who participated in the present study. Excepting the Sensory Profile 2, our results were similar to those reported in other studies conducted in different countries and which mainly focused on assessing cognitive impairment [16,19,23,24,30]. In the fields of neurology and geriatrics, functional assessment tools, such as the Barthel Index, Lawton Instrumental ADL Scale and FIM were found to be the most used measures in occupational therapy practice [23]. As mentioned above, the greater use of these assessment tools observed in our study could mainly be explained by the fact that more than half of the participants treated people affected by neurological disorders or neurodegenerative diseases at the time of the survey. However, it was also observed that, compared to the other studies referred to, the occupational therapists participating in the present study used the Lawton Scale more often. According to a recent systematic review of ADL assessment tools [31], the number of IADL instruments is limited and, as far as we know, the Lawton scale is currently the only measure available and/or validated for use in the Spanish population. Concerning the results obtained for the Sensory Profile 2, around a seventh of the occupational therapists provided early intervention services focused on children, and some worked in schools. Although several tools are available in Spanish for assessing sensory processing, the Sensory Profile (either the first or second edition) is one of the most widely used, especially in children [25,32]. Moreover, the recent validation of the most recent Spanish version of Sensory Profile 2 could also partly explain its wider use among Spanish occupational therapists [33].

An important observation in the present study is that around a third of the participants reported having doubts about, or not knowing, whether the tools they used were valid for the Spanish population. This finding is similar to that obtained in other studies, in which 40–50% of occupational therapists reported using assessment tools but were unaware of whether the measures were standardised and/or valid [16,23]. Although the reasons why participants could not identify the scientific validity of the measures were not collected, lack of knowledge about the appropriateness of the assessment tool is a possible explanation. This may be because occupational therapists usually select a tool depending on its “availability at the workplace”, “quickness to administer”, and/or “ease of interpretation” [16] rather than its accuracy. As using standardised instruments is inherent to evidence-based practice and requires research expertise, another plausible reason might be the lack of research knowledge and skills to choose a valid and reliable assessment tool. In the case of occupational therapists, evidence shows that lack of confidence and skills in appraising research is a common reason that prevents them from applying evidence-based practice in their clinical practice [34,35,36,37,38]. Thus, it is reasonable to suppose that ignorance about the scientific properties of an instrument could be because many occupational therapists who participated in this study may have difficulty in using adequate and reliable evidence in their practice, mainly because of a lack of research training. Regrettably, research competence needed for evidence-based practice is still poorly integrated into healthcare program curriculum design, including education programs for occupational therapy [39,40]. In this respect, more initiatives are needed to improve evidence-based practice and clinical effectiveness in occupational therapy.

Similarly, although further exploration is required, the findings of this study also appear to indicate that using non-standardised tools in occupational therapy practice could be more extensive than expected. In this sense, considering the most frequently used assessment tools in occupational therapy are limited to a few instruments, it may be suggested that occupational therapists use their own ad hoc measures for assessment during their routine clinical practice rather than standardised measures. Finally, a further suggestion is that the small number of performance-based occupational assessments currently available in Spanish represents a significant limiting factor in the use and knowledge of standardised assessment tools.

This study has some limitations. Firstly, the small number of participants in this study may make the sample non-representative, limiting the generalisability of the findings. In addition, the fact that the questionnaire was disseminated via social networks and email may have hindered the inclusion of occupational therapists who do not use social media. However, the observed prevalence of use of assessment tools in occupational therapy practice according to the practice setting or clinical area was similar to that found in previous studies conducted in different countries [16,17,18,19,20,21,22,23,24,25]. Another limitation is that the self-reported questionnaire used to collect the information about the assessment tools may involve a recall bias. The participants are likely not to have reported some assessment tools because they did not use them often in their clinical practice. However, we identified many potential measures that can be used in the evaluation process of occupational therapy. Moreover, the most used assessment tools identified in this study were similar to those reported in other studies. 

Despite its potential shortcomings, this study may contribute to the understanding of current trends in occupational therapy practice by accumulating evidence about the use of assessment tools. Moreover, this is the first time a study has examined the use and type of assessment tools in Spanish occupational therapists. Apart from the large amount of helpful information provided, this study has the additional advantage of classifying assessment tools into the domains of occupational therapy, which will enable us to plan future initiatives specifically aimed at clinicians and researchers in occupational therapy. In this respect, as exploratory research, this study may constitute a positive step forward for developing strategies to enhance evidence-based assessment in occupational therapy practice.

## 5. Conclusions

This study showed that the most frequently used assessment tools in occupational therapy in Spain were instruments for measuring ADL, including IADL, body functions, process skills, and motor skills. More specifically, the Barthel Index, Lawton Instrumental ADL Scale, FIM, Sensory Profile 2 and MEC were found to be the most used assessment tools amongst Spanish occupational therapists. These findings align with earlier studies, especially those focusing on occupational therapists working with neurological disorders. However, these results also indicate that evidence-based assessment in occupational therapy remains challenging. Evidence-based assessment using standardised measures is essential to ensure high-quality healthcare delivery and patient safety as a critical element in clinical decision-making. In this sense, more efforts are needed to increase the use and knowledge of standardised tools in occupational therapy. Furthermore, in line with recent initiatives aimed at enhancing research skills training for Spanish-speaking occupational therapists [41], this study’s findings can contribute to the development of new strategies for improving evidence-based assessment in occupational therapy. It is hoped that this study may serve as a basis for future research focused on exploring the factors related to evidence-based practice in the field of occupational therapy, including the use of standardised assessment measures.

## Figures and Tables

**Table 1:** General characteristics of the study participants (*n* = 73).

Variables	
Age, median (IQR)	31 (26–37)
Sex, *n* (%)	
Female	63 (86.3)
Male	10 (13.7)
Occupational therapy degree ^1^, *n* (%)	
3-year bachelor’s degree	36 (52.2)
4-year bachelor’s degree	33 (47.8)
Date of completion of occupational therapy degree ^1^, *n* (%)	
<2009	22 (33.3)
≥2009	44 (66.7)
Master’s degree ^1^, *n* (%)	
Yes	47 (69.1)
No	21 (30.9)
Employment status ^1^, *n* (%)	
Employed	57 (78.1)
Self-employed	11 (15.1)
Unemployed	4 (5.5)
Weekly working hours, median (IQR)	34 (20–40)
Workplace, *n* (%)	
Private clinic	28 (38.4)
Public health centre	6 (8.2)
Foundation/Association	18 (24.7)
Occupational centre	2 (2.7)
Client’s home	1 (1.4)
University	7 (9.6)
Others	11 (15.1)
Practice setting	
Neurology	28 (38.4)
Dementia	9 (12.3)
Geriatrics	8 (11.1)
Early childhood intervention	9 (12.3)
Mental health	5 (6.9)
Community care	2 (2.7)
Intellectual disability	3 (4.1)
School	2 (2.7)
Academic teaching/research	2 (2.7)
Traumatology/rheumatology	2 (2.7)
Others	3 (4.1)

Abbreviations: IQR, interquartile range. ^1^ Data available for occupational therapy degree (*n* = 69); Date of completion of occupational therapy degree (*n* = 66); Master’s degree (*n* = 68); Employment status (*n* = 72).

**Table 2:** Percentage use of 147 assessment tools reported by the participants according to the main domains of occupational therapy based on the Occupational Therapy Practice Framework.

Domains of Occupational Therapy *	*n*	%
Occupations		
Activities of daily living	56	76.7
Instrumental activities of daily living	45	61.6
Health management	15	20.5
Rest and sleep	5	6.8
Education	12	16.4
Work	13	17.8
Play	11	15.1
Leisure	13	17.8
Social participation	21	28.8
Contexts		
Environmental factors	8	11.0
Personal factors	8	11.0
Performance patterns		
Habits	9	12.3
Routines	10	13.7
Rituals	8	11.0
Roles	8	11.0
Performance skills		
Motor skills	47	64.4
Process skills	57	78.1
Social interaction skills	41	56.2
Client Factors		
Values, beliefs, and spirituality	7	9.6
Body functions	62	84.9
Body structures	7	9.6
Outcomes		
Quality of life	1	1.4
Health and wellness	1	1.4
Well-being	4	5.5

* Classification according to the fourth edition of the American Occupational Therapy Association framework [26].

**Table 3:** Ranking of the ten assessment tools most frequently used by the study participants.

Assessment Tool	Used		Validated Tool ^1^		Domains of Occupational Therapy ^2^
	n	%	n	%	
Barthel Index	37	50.7	30	81.8	Occupations: Activities of daily living
Lawton Instrumental ADL Scale	29	39.7	20	68.7	Occupations: Instrumental activities of daily living
Functional Independence/Assessment Measure	16	21.9	13	81.2	Occupations: Activities of daily living, Instrumental activities of daily living; Performance Skills: Process skills, social interaction skills; Client Factors: Body functions
Sensory Profile 2	15	20.5	6	40.0	Client Factors: Body functions
Mini-Examen Cognoscitivo	11	15.1	9	81.8	Performance Skills: Process skills; Client Factors: Body functions
Sensory Integration and Praxis Test	10	13.7	2	20.0	Performance Skills: Motor skills, process skills; Client Factors: Body functions
Mini-Mental State Examination	9	12.3	6	66.7	Performance Skills: Process skills; Client Factors: Body functions
Loewenstein Occupational Therapy Cognitive Assessment	9	12.3	5	55.5	Performance Skills: Motor skills, process skills; Client Factors: Body functions
Nine-Hole Peg Test	8	11.0	7	87.5	Performance Skills: Motor skills; Client Factors: Body functions
Tinetti Test	8	11.0	3	37.5	Performance Skills: Motor skills; Client Factors: Body functions

Abbreviations: ADL, activities of daily living. ^1^ Frequencies (*n* and %) of participants that reported the tools they used were validated. ^2^ Classification according to the fourth edition of the American Occupational Therapy Association framework [26].

## Data Availability

All the study data will be available to interested researchers upon request to E.-M.N.-M., who is responsible for the study. Requests will be reviewed by the research team and will require a data transfer agreement.

## Supplementary Materials

The following supporting information can be downloaded at: https://www.mdpi.com/article/10.3390/healthcare10101902/s1, Supplementary Materials S1: List of assessment tools.
