# Supplementary material for: Identification and Use of Assessment Tools in Spanish Occupational Therapists: An Exploratory Study
Source: Healthcare (Basel). 2022 Sep 28;10(10):1902. doi: 10.3390/healthcare10101902 (PMC9601930; doi:10.3390/healthcare10101902)
Supplement: Supplementary file 1 [file healthcare-10-01902-s001.zip › healthcare-1858087-supplementary.pdf]

Supplementary Materials

S1. List of 147 assessment tools that 73 occupational therapists reported using

**Assessment Tool**	**Occupational Therapy Domains**	**Used, *n***	**Validated tool*, *n***
Agitated Behavior Scale (ABS)	Performance Skills: Social interaction skills; Client Factors: Body functions	1	1
ABILHAND-Kids	Performance Skills: Motor skills	1	0
Action Research Arm Test (ARAT)	Performance Skills: Motor skills; Client Factors: Body functions	1	0
Adaptive Behavior Assessment System (ABAS)	Occupations: ADLs, IADLs, Health management, Education, Work, Leisure, Social Participation; Performance Skills: Process skills, Social interaction skills	1	0
Adaptive Behavior Assessment System-Second Edition (ABAS-II)	Occupations: ADLs, IADLs, Health management, Education, Work, Leisure, Social Participation; Performance Skills: Process skills, Social interaction skills	1	1
Ages & Stages Questionnaires (ASQ-3)	Performance Skills: Motor skills, Process skills, Social interaction skills; Client Factors: Body functions	4	2
Algometry	Client Factors: Body functions	1	0
Alzheimer disease assessment scale-cognitive (ADAS-COG)	Performance Skills: Process skills, Social Interaction skills; Client Factors: Body functions	1	0
Apraxia Screen of Tulia (AST)	Performance Skills: Motor skills, Process skills, Social interaction skills	2	1
Assessment of Communication and Interaction Skills (ACIS)	Performance Skills: Social interaction skills	2	0
Assessment of Motor and Process Skills (AMPS)	Performance Skills: Motor skills, Process skills	3	1
Attention Deficit Hyperactivity Disorder Assessment Scale (EDAH)	Performance Skills: Process skills, Social Interaction skills; Client Factors: Body functions	1	1
Ayres Sensory Integration (EASI)	Performance Skills: Motor skills, Process skills, Social interaction skills; Client Factors: Body functions	3	1
Barcelona Test	Performance Skills: Process skills, Social Interaction skills; Client Factors: Body functions	2	2
Baremo de Valoración de la Dependencia (BVD)	Occupations: ADLs, IADLs, Health management; Performance Skills: Motor skills	1	0
Barthel Index	Occupations: ADLs	37	30
Basic Everyday Living Schedule (BELS)	Occupations: ADLs, IADLs, Health management, Leisure, Social participation	1	1

Battelle Developmental Inventory	Performance Skills: Motor skills, Process skills, Social interaction skills	3	1
Batterie d'évaluation Talbot	Occupations: ADLs; Performance Skills: Motor skills, Process skills, Social interaction skills; Client Factors: Body functions	2	1
Beck Depression Inventory-Second Edition (BDI-II)	Client Factors: Body functions	1	1
Beery-Buktenica Developmental Test of Visual-Motor Integration (VMI)	Performance Skills: Motor skills, Process skills; Client Factors: Body functions	2	2
Behavior Rating Inventory of Executive Function-Second Edition (BRIEF-2)	Performance Skills: Process skills, Social interaction skills; Client Factors: Body functions	1	0
Berg Balance Scale	Performance Skills: Motor skills; Client Factors: Body functions	1	0
Box and Blocks Test	Performance Skills: Motor skills; Client Factors: Body functions	5	2
Bruininks-Oseretsky Test (BOT)	Performance Skills: Motor skills; Client Factors: Body functions	1	0
Brunet-Lezine Test	Performance Skills: Motor skills, Process skills	2	2
California Verbal Learning Test (CVLT)	Performance Skills: Process skills; Client Factors: Body functions	1	0
Canadian Occupational Performance Measure (COPM)	All aspects of the domain	3	3
Chessington Occupational Therapy Neurological Battery (COTNAB)	Performance Skills: Process skills, Social interaction skills; Client Factors: Body functions	5	1
Childhood Autism Rating Scale (CARS)	Performance Skills: Motor skills, Process skills, Social interaction skills; Client Factors: Body functions	1	0
Children's Hand-use Experience Questionnaire (CHEQ)	Performance Skills: Motor skills	1	1
Coin Test-EUROTEST	Performance Skills: Process skills; Client Factors: Body functions	1	0
Coma Recovery Scale-Revised	Performance Skills: Process skills; Client Factors: Body functions	1	0
Comprehensive Observations of Proprioception	Performance Skills: Motor skills; Client Factors: Body functions	3	1
Comprehensive Occupational Therapy Evaluation Scale (COTE)	Performance Skills: Motor skills, Process skills, Social interaction skills; Client Factors: Body functions	1	1
Computarized Dynamic Posturography	Performance Skills: Motor skills; Client Factors: Body functions	1	0
Daniels Scale	Client Factors: Body functions	3	3
DeGangi-Berk Test of Sensory Integration (TSI)	Performance Skills: Motor skills; Client Factors: Body functions	2	2
Delirium Rating Scale (DRS)	Performance Skills: Process skills; Client Factors: Body functions	1	1

Denver Test	Performance Skills: Motor skills, Process skills, Social interaction skills; Client Factors: Body functions	1	0
Developmental profile	Occupations: ADLs, IADLs; Performance Skills: Motor skills, Process skills, Social interaction skills; Client Factors: Body functions	1	0
Disability assessment for dementia (DAD)	Occupations: ADLs, IADLs; Health management	1	1
Duruöz Hand Index	Performance Skills: Motor skills	1	0
Dynamic Occupational Therapy Cognitive Assessment for Children (DOTCA-Ch)	Performance Skills: Motor skills, Process skills; Client Factors: Body functions	1	0
Dynamometer	Client Factors: Body functions	3	2
Early Development Inventory	Occupations: Play; Performance Skills: Motor skills, Process skills; Client Factors: Body functions	1	0
Escala de incapacidad física de la Cruz Roja	Occupations: ADLs, IADLs; Performance Skills: Process skills, Social interaction skills; Client Factors: Body functions	1	1
Escalas del Hospital de Sagunto	Performance Skills: Motor skills; Client Factors: Body functions	1	0
Esthesiometry	Client Factors: Body functions	1	0
Family Impact Questionnaire (FIQ)	Outcomes: Well-being	1	0
Fatigue Severity Scale (FSS)	Performance Skills: Motor skills; Client Factors: Body functions	1	0
Free Times Test (FTT)	Occupations: Leisure	1	0
Frontal Assessment Battery (FAB)	Performance Skills: Process skills; Client Factors: Body functions	2	2
Functional Independence/Assessment (FIM+FAM)	Occupations: ADLs, IADLs; Performance Skills: Process skills, Social interaction skills; Client Factors: Body functions	16	13
Functional Reach Test	Performance Skills: Motor skills; Client Factors: Body functions	1	0
Galveston Orientation and Amnesia Test (GOAT)	Client Factors: Body functions	1	1
GENCAT Scale	Outcomes: Quality of life	1	0
Global Deterioration Scale de Reisberg (GDS)	Performance Skills: Process skills, Social interaction skills; Client Factors: Body functions	2	2
Goldberg Depression Questionnaire	Client Factors: Body functions	1	1
Goniometry	Client Factors: Body functions	3	3
Gross Motor Function Measure (GMFM)	Performance Skills: Motor skills; Client Factors: Body functions	2	1
Harris Test	Client Factors: Body functions	1	0

Home Observation for Measurement of the Environment (HOME)	Contexts: Environmental factors	1	0
Informant Interview	Occupations: ADLs; Performance Skills: Process skills; Client Factors: Body functions	1	1
Interest Checklist	Occupations: Education, Work, Play, Leisure, Social participation	1	0
Inventario de Espectro Autista (IDEA)	Performance Skills: Process skills, Social interaction skills; Client Factors: Body functions	1	0
Isaac Set-Test	Client Factors: Body functions	2	2
Jebsen-Taylor Hand Function Test (JTHFT)	Performance Skills: Motor skills; Client Factors: Body functions	1	1
Katz Index of Independence in Activities of Daily Living	Occupations: ADLs	1	1
Kinesthetic and Visual Imagery Questionnaire (KVIQ)	Performance Skills: Motor skills; Client Factors: Body functions	1	0
Lawton Instrumental Activities of Daily Living Scale (IADL)	Occupations: IADLs	29	20
Leisure Interest Assessment	Occupations: Leisure	1	0
Loewenstein Occupational Therapy Cognitive Assessment (LOTCA)	Performance Skills: Motor skills, Process skills; Client Factors: Body functions	9	5
Loewenstein Occupational Therapy Cognitive Assessment-Geriatric (LOTCA-G)	Performance Skills: Motor skills, Process skills; Client Factors: Body functions	1	0
Manual Ability Classification System (MACS)	Performance Skills: Motor skills; Client Factors: Body functions	1	0
Measures of Academic Progress (MAP)	Performance Skills: Process skills, Client Factors: Body functions	1	0
Miller Assessment for Preschoolers	Performance Skills: Motor skills, Process skills, Social interaction skills; Client Factors: Body functions	1	1
Mini Examen Cognoscitivo de Lobo (MEC)	Performance Skills: Process skills; Client Factors: Body functions	11	9
Mini-Mental State Examination (MMSE)	Performance Skills: Process skills; Client Factors: Body functions	9	6
Minnesota Multiphasic Personality Inventory (MMPI)	Client Factors: Body functions	2	0
Model of Human Occupation Screening Tool (MOHOST)	Occupations: Social participation; Performance Skills: Process skills, Social interaction skills; Client Factors: Body functions	2	0
Modified Ashworth Scale	Client Factors: Body functions	1	1
Modified Interest Checklist	Occupations: Education, Work, Play, Leisure, Social participation	1	1
Montreal Cognitive Assessment (MoCA)	Performance Skills: Process skills; Client Factors: Body functions	1	1
Motor Activity Log	Performance Skills: Motor skills	1	1

Motor Coordination Test 3JS	Performance Skills: Motor skills; Client Factors: Body functions	1	0
Movement Assessment Battery for Children (MACB)	Performance Skills: Motor skills; Client Factors: Body functions	3	2
Movement Assessment Battery for Children- Second Edition, (MACB-2)	Performance Skills: Motor skills; Client Factors: Body functions	1	1
Movement Imagery Questionnaire (VMIQ)	Performance Skills: Motor skills; Client Factors: Body functions	1	1
Nine Hole Peg Test	Performance Skills: Motor skills; Client Factors: Body functions	8	7
Nottingham Sensory Assessment	Client Factors: Body functions	2	1
Occupational Profile	Occupations: ADLs, IADLs, Health management, Rest and sleep, Education, Work, Play, Leisure, Social Participation; Contexts: Environmental and personal factors; Performance Patterns: Habits, Routines, Roles, Rituals; Client Factors: Values, beliefs, and spirituality, Body functions, Body structures	1	0
Occupational profile and history (Isabel Beaudry's adaptation)	Occupations: ADLs, IADLs, Rest and sleep, Work, Play, Leisure, Social participation; Contexts: Environmental and personal factors; Performance Patterns: Habits, Routines, Roles, Rituals; Performance Skills: Social interaction skills; Client Factors: Body functions, Body structures	1	0
Occupational Self-Assessment (OSA)	Occupations: ADLs, IADLs, Health management, Education, Work, Social participation; Contexts: Environmental and personal factors; Performance Patterns: Habits, Routines, Roles; Performance Skills: Process skills, Social interaction skills	1	0
OPHI-II Interview	Occupations: Health management, Leisure, Social participation; Contexts: Environmental and personal factors; Performance Patterns: Habits, Routines, Roles; Client Factors: Values, beliefs, and spirituality, Body functions	3	0
Pain visual analogue scale	Client Factors: Body functions	2	1
Parenting Stress Index (PSI)	Outcomes: Well-being	1	1
Peabody Developmental Motor Scales-Second Edition (PDMS-2)	Performance Skills: Motor skills; Client Factors: Body functions	2	0
Pediatric Evaluation of Disability Inventory (PEDI)	Occupations: ADLs, Play, Social Participation; Performance Skills: Motor skills, Social interaction skills; Client Factors: Body functions	3	2
Pinch Grip Test	Client Factors: Body functions		

Play History (PH)	Occupations: Play	1	0
Portrait Values Questionnaire (PVQ)	Client Factors: Values, beliefs, and spirituality	1	0
Postural Assessment Scale for Stroke (PASS)	Performance Skills: Motor skills; Client Factors: Body functions	1	0
Preschool Play Scale (PPS)	Occupations: Play, Social participation; Performance Skills: Motor skills, Process skills, Social interaction skills; Client Factors: Body Functions	1	0
Purdue Pegboard Test (PPT)	Performance Skills: Motor skills; Client Factors: Body functions	4	1
Quick Dash Test	Performance Skills: Motor skills; Client Factors: Body functions	1	0
Rivermead Behavioural Memory Test (RBMT)	Performance Skills: Process skills; Client Factors: Body functions	2	0
Routine Task Inventory (RTI)	Occupations: ADLs, IADLs; Performance Skills: Process skills, Social interaction skills	1	0
Routines-Based Interview	Performance Patterns: Habits, Routines, Roles	8	0
Screen for Cognitive Impairment in Psychiatry (SCIP-S)	Performance Skills: Process skills, Social interaction skills; Client Factors: Body functions	1	1
Screening Neuropsicológico para Niños (SNN-UBA)	Performance Skills: Process skills, Social interaction skills; Client Factors: Body functions	1	0
Semantic Verbal Fluency test (SVF)	Performance Skills: Social interaction skills; Client Factors: Body functions	1	1
Semmes-Weinstein monofilament test	Client Factors: Body functions	2	1
Sensory Integration and Praxis Test (SIPT)	Performance Skills: Motor skills, Process skills; Client Factors: Body functions	10	2
Sensory Processing Measure (SPM)	Performance Skills: Motor skills, Process skills, Social interaction skills; Client Factors: Body functions	3	0
Sensory Profile	Client Factors: Body functions	1	1
Sensory Profile-Second Edition	Client Factors: Body functions	15	6
Short Child Occupational Profile	Occupations: ADLs, Social participation; Performance Skills: Motor skills, Process skills, Social interaction skills; Client Factors: Body functions	1	1
Short Portable Mental State Questionnaire (SPMSQ)	Client Factors: Body functions	5	5
Short Sensory Profile	Client Factors: Body functions	1	1
Short-wavelength automated perimetry (SWAP)	Client Factors: Body functions	1	0
Social Functioning Scale (SFS)	Occupations: ADLs; IADLs, Work, Leisure, Social participation;	1	1

	Performance Skills: Social interaction skills		
Stages of Recovery Instrument (STORI)	Outcomes: Well-being, Health and wellness	1	1
Star Cancellation Test	Performance Skills: Process skills; Client Factors: Body functions	1	0
Stroke Rehabilitation Assessment of Movement (STREAM)	Performance Skills: Motor skills; Client Factors: Body functions	1	0
Structured Observations of Sensory Integration-Motor (SOSI-M)	Performance Skills: Motor skills, Process skills; Client Factors: Body functions	2	1
Stuttering Severity Instrument (SSI)	Client Factors: Body functions	1	0
Supports Intensity Scale (SIS)	Occupations: ADLs, IADLs, Health management, Work, Leisure, Social participation	1	0
Test de Análisis de Lectoescritura (TALE)	Performance Skills: Social interaction skills, Client Factors: Body functions	1	0
Test of Sensory Functions in Infants (TSFI)	Performance Skills: Motor skills; Client Factors: Body functions	5	1
The Checklist of Adaptive Living Skills (CALS)	Occupations: ADLs, IADLs, Health management, Work, Leisure, Social participation; Performance Skills: Social interaction skills	2	0
The Child Occupational Self-Assessment (COSA)	Occupations: ADLs, IADLs, Education, Social participation; Performance skills: Motor skills, Social participation skills; Client Factors: Body functions	1	0
The clock-drawing test	Performance Skills: Process skills; Client Factors: Body functions	2	1
The Modified Checklist for Autism in Toddlers (M-CHAT)	Performance Skills: Motor skills, Process skills, Social interaction skills; Client Factors: Body Functions	8	0
The Role Checklist	Performance Patterns: Roles	1	0
Tinetti-test	Performance Skills: Motor skills; Client Factors: Body functions	8	3
Trail Making Test (TMT)	Performance Skills: Process skills; Client Factors: Body functions	1	1
Unstructured and Structured Observations of Sensory Integration	Performance Skills: Motor skills, Process skills; Client Factors: Body Functions	1	0
Variable Handling Assessment Charts (VMAC)	Occupations: Health management, Work	1	0
VertiGuard system	Performance Skills: Motor skills; Client Factors: Body functions	1	0
Vineland Social Maturity Scale	Occupations: ADLs, IADLs, Play, Social Participation; Performance Patterns: Routines; Performance Skills: Motor skills, Process skills, Social interaction skills; Client Factors: Body Functions	1	0

Volitional Questionnaire (VQ)	Outcomes: Well-being	2	0
Volumetric assessment	Client Factors: Body functions	1	1
Wee - Functional Independence Measure (WeeFIM)	Occupations: ADLs, IADLs, Social participation; Performance Skills: Process skills, Social interaction skills; Client Factors: Body Functions	2	0
WHODAS	Occupations: ADLs, IADLs, Education, Work, Social Participation; Performance Skills: Motor skills, Social interaction skills; Client Factors: Body Functions	1	1
Wolf Motor Function Test (WMFT)	Performance Skills: Motor skills; Client Factors: Body functions	1	1
Yesavage Geriatric Depression Scale	Client Factors: Body functions	2	1

* Number of tools that the occupational therapists reported using and identified as validated assessment tools.

S2. Online survey

Section 1. Assessment tools

1. Indicate the assessment tools that you use during your professional practice. Please, indicate the full name whenever possible:
2. Which of the assessment tools mentioned above do you know that are adapted and validated for the Spanish population?
3. Indicate other assessment tools that you know. Please, indicate the full name whenever possible:
4. Which of the assessment tools mentioned above do you know that are adapted and validated for the Spanish population?

Section 2. Socio-demographic information

5. Age:	
6. Sex:	
	Male
	Female
7. Employment status:	
	Self-Employed
	Employed
	Unemployed
	Other
8. Zip Code of the workplace where you are working at this moment:	
9. Current workplace. Please, indicate more than one if necessary:	
	Private clinic
	Public health center
	Educational center
	Occupational center
	Foundation/Association
	Insurance company
	Client's home
	Supervised apartments
	Orthopedic aids stores
	University
	Other:
10. Current practice setting. Please, indicate more than one if necessary:	
	Neurology
	Traumatology/Rheumatology
	Mental health
	Dementia
	Early childhood intervention
	Rare diseases
	Drug dependences
	Intellectual disabilities
	School
	Education/Academic teaching
	Research
	Community care
	Assistive products
	Burn injuries
	Geriatrics
	Other:
11. Please, indicate the number of hours you work per week:	

Section 3. Academic background

12. Indicate the university name where you studied:	
13. Occupational therapy studies:	
	Diploma in Occupational Therapy
	Degree in Occupational Therapy
14. Have you studied for a master's degree?	
	Yes
	No
15. Please, indicate the master's name:	
16. Indicate the date of completion of occupational therapy degree:	
